# Transmission of HIV-1 CTL Escape Variants Provides HLA-Mismatched Recipients with a Survival Advantage

**DOI:** 10.1371/journal.ppat.1000033

**Published:** 2008-03-21

**Authors:** Denis R. Chopera, Zenda Woodman, Koleka Mlisana, Mandla Mlotshwa, Darren P. Martin, Cathal Seoighe, Florette Treurnicht, Debra Assis de Rosa, Winston Hide, Salim Abdool Karim, Clive M. Gray, Carolyn Williamson, and the CAPRISA 002 Study Team

**Affiliations:** 1 Institute of Infectious Diseases and Molecular Medicine, Division of Medical Virology, Faculty of Health Sciences, University of Cape Town, Cape Town, South Africa; 2 Centre for the AIDS Programme of Research in South Africa, University of Kwa-Zulu Natal, Durban, South Africa; 3 National Institute for Communicable Diseases, Johannesburg, South Africa; 4 South African National Bioinformatics Institute, University of the Western Cape, Cape Town, South Africa; National Institutes of Health-NIAID, United States of America

## Abstract

One of the most important genetic factors known to affect the rate of disease progression in HIV-infected individuals is the genotype at the Class I Human Leukocyte Antigen (HLA) locus, which determines the HIV peptides targeted by cytotoxic T-lymphocytes (CTLs). Individuals with HLA-B*57 or B*5801 alleles, for example, target functionally important parts of the Gag protein. Mutants that escape these CTL responses may have lower fitness than the wild-type and can be associated with slower disease progression. Transmission of the escape variant to individuals without these HLA alleles is associated with rapid reversion to wild-type. However, the question of whether infection with an escape mutant offers an advantage to newly infected hosts has not been addressed. Here we investigate the relationship between the genotypes of transmitted viruses and prognostic markers of disease progression and show that infection with HLA-B*57/B*5801 escape mutants is associated with lower viral load and higher CD4+ counts.

## Author Summary

Following infection with HIV, it is well established that a person's genetic makeup is a major determinant of how quickly they will progress to AIDS. Particularly important is the class I Human leukocyte antigen (HLA) gene that is responsible for alerting the immune system to HIV's presence. One of the reasons our immune systems are unable to beat HIV is that the virus can mutate to forms that our HLA genes no longer recognise. However, some people have versions of the HLA gene (for example HLA-B*57 and HLA-B*5801) that are known to force HIV to tolerate mutations that damage its ability to reproduce. Slower HIV reproduction is thought to be one reason that HLA-B*57 and HLA-B*5801 positive people progress to AIDS more slowly than most other HIV infected persons. We report here on a study of HLA-B*57 and HLA-B*5801 negative women in which better control of disease tended to be associated with their being infected with viruses carrying mutations that have been previously shown to reduce replication. These mutations characterise viruses found infecting HLA-B*57 and HLA-B*5801 positive people. This indicates for the first time that HLA-B*57 or HLA-B*5801 negative people that are infected by such reproductively compromised viruses may also experience better survival prospects.

## Introduction

Avoidance of host anti-viral responses is a major factor influencing the evolution of HIV genomes. Of particular importance for virus survival, and a major contributor to ongoing HIV-1 diversification worldwide, is continual escape from anti-HIV host cytotoxic T lymphocyte (CTL) responses. CTLs can potentially detect many small polypeptide epitope sequences encoded throughout HIV genomes. Evasion of CTL responses involves mutations within and around targeted epitopes that result in the peptide no longer binding to the Class I MHC grove, or non-recognition by the CTL T cell receptor, or interference with peptide processing [1]–[5]. These so-called CTL escape mutations have been associated with increased viral loads and more rapid disease progression [3], [6]–[8]. However, mutations associated with CTL evasion can also incur significant viral replicative fitness costs and some escape mutations have therefore been associated with decreased viral loads. In the macaque model, for example, *in vitro* replication of SIVmac239 variants carrying certain CTL escape mutations is impaired relative to SIVmac239 without the mutations [9],[10]. Fitness costs associated with CTL escape have also been demonstrated in HIV-1 infected humans carrying either the B*57 or B*5801 HLA alleles. CTL escape mutations that frequently arise in these individuals, such as the T242N mutation in the Gag TW10 epitope and the A163X (X = G, N, D or S) mutation in the KF11 epitope, have been found to compromise viral replicative capacity [11],[12].

Because of the fitness costs associated with CTL escape mutations, specific HLA alleles backgrounds of HIV infected individuals have an influence on rates of disease progression. For example, HIV-infected individuals possessing the B*57, B*5801 and B*27 HLA-alleles tend to take significantly longer to progress to AIDS than individuals without these alleles [13]–[16]. HIV-1 epitopes targeted by these HLA types occur within functionally important protein domains and escape mutations in these domains tend to decrease viral replicative fitness [9], [11], [12], [17]–[19]. Therefore decreased rates of disease progression in people carrying the B*57, B*5801 and B*27 alleles is at least partially driven by HLA associated virus attenuation.

From the virus' perspective, the conflicting demands of replicative fitness on one hand and immune evasion on the other, are best illustrated by the high rates at which certain CTL escape mutations revert to ancestral, presumably replicationally fitter, states following transmission to HLA-mismatched hosts [12],[20],[21]. Whenever CTL-escape mutations do not revert following transmission to such hosts it is generally assumed either that the fitness costs of the mutations are negligible [21],[22], or that the replicative fitness of escape mutants has been effectively restored by compensatory mutations [11],[12],[17],[21],[23],[24].

While much effort has been focused on demonstrating the causal influence of host genetic features on reduced viral replication and decreased rates of disease progression [7], [25]–[28], there are a few instances where viral genetic features alone have been identified as the primary cause of slower disease progression. For example, a study dealing with individuals infected with contaminated blood from a common donor determined that long term non-progression was due to transmitted HIV genomes carrying deletions in *nef* and the terminal repeat region [29],[30].

Here we describe the identification of two HIV-1 Gag polymorphisms that are associated with low viral loads and high CD4+ counts during both the acute and chronic phases of infection. Although these polymorphisms have been previously identified as attenuating CTL escape mutations in individuals carrying either the HLA-B*57 or B*5801 alleles, we detect these associations in a group of HLA-B*57/5801-negative individuals. We propose that these “attenuating” polymorphisms probably arose during virus passage through HLA-B*57/5801-positive individuals and provide evidence that they have enabled better control of viral replication for up to at least a year following transmission to their current hosts. This is the first demonstration that transmission of HIV variants carrying HLA-associated escape mutations may also afford improved control of virus replication in HLA-mismatched recipients. Our results suggest a dependency between the rate of disease progression in the newly infected host and the genotype of the individual from whom the virus was acquired.

## Results

HLA-B*27, -B*57 and -B*5801 alleles are associated with long term non-progression [13]–[16], and participants with these HLAs were therefore excluded from this investigation. Of an initial twenty-four study participants enrolled, three were HLA B*5801 positive and none were HLA-B*27, -B*57 positive. The remaining twenty-one HLA-B*27, -B*57 and -B*5801 negative individuals, estimated to be between 22 and 62 days post HIV infection (median = 42 days) at their time of enrollment, were recruited and followed-up for at least 12 months (Table S1). The median log viral load and CD4+ count went from 4.71 copies.ml^−1^ (range 2.95 to 6.28) and 509 cells.ul^−1^ (range 255 to 1358), respectively, at three months post-infection to 4.59 copies.ml^−1^ (range 2.60 to 6.09) and 367 cells.ul^−1^ (range 202 to 1030), at twelve months. For each participant, complete *gag* genes were amplified and sequenced from the earliest available HIV positive sample, and samples taken at 3 and 6 months postinfection.

### Gag amino acids 146 and 242 are associated with control of virus replication

We tested for statistical association between polymorphic amino acid positions and both viral load and CD4+ cell counts. This identified amino acid polymorphisms at two sites in Gag, at HXB2 positions 146 (n = 9) and 242 (n = 6), that were associated with higher than average CD4+ counts and lower than average viremia (Table 1). Nine of the 21 study participants were infected with viruses carrying the A146X (X = P, or S) polymorphism and the viruses in six of these nine individuals also carried the T242N mutation. Gag amino acid 146 is adjacent to the HLA-B*57/5801 restricted ISW9 epitope and the A146X polymorphism has previously been identified as an epitope processing mutation associated with CTL escape [22] (Table 1). Similarly, position 242 occurs in the immunodominant TW10 epitope and the T242N polymorphism has also previously been associated with CTL escape in HLA-B*57/5801 positive individuals [21]. We, therefore suspected that viruses carrying either one or both of these two polymorphisms may have been CTL escape variants that had been transmitted from HLA-B*57/5801 positive individuals. Whereas *in vitro* studies have shown that the A146X mutation does not incur a replicative fitness cost, the T242N is known to decrease viral fitness [11],[22]. There was only marginal statistical significance (p = 0.0733) for an association between the presence of both mutations (T242N/A146X) (n = 6) and lower viral loads, however when three additional infections involving viruses carrying the A146X mutation only were included in the analysis (n = 9), the association was strengthened (p = 0.0275), suggesting that the T242N mutation is not solely responsible for the association (Table 1).

**Table 1:** The identification of sites associated with high CD4+ counts and low viral loads at 12 months post infection.

			Median CD4+ count^a^			Median log_10_ viral load^a^		
Mutation	n	Epitope	+	-	p-value	+	-	p-value
A146X	9	ISW9	544	348	0.0172	3.49	4.87	0.0275
T242N	6	TW10	538	390	0.0175	3.26	4.69	0.0733

a (+) Presence of mutation; (−) absence of mutation

### T242N and A146X mutations are consistent with transmission from B*57/B*5801 positive individuals

**Figure 1:**
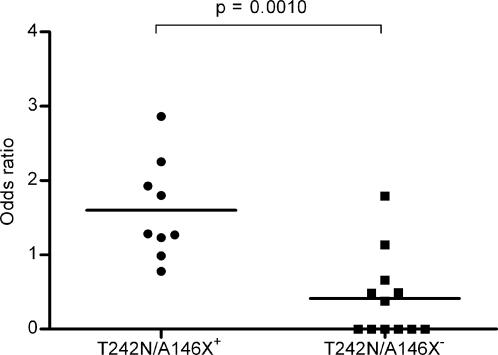
The accumulation of non-synonymous mutations within the three B57/B5801-specific immunodominant epitopes. Comparison of the odds ratio of non-synonymous mutations within TW10, ISW9 and KF11 between those B57/B5801-negative participants infected with variants carrying the T242N and A146X mutations and those not carrying the mutations.

If viruses carrying the T242N and A146X mutations were transmitted from either B*57 or B*5801 positive donors, we hypothesised that selection should be evident at sites within immunodominant B*57 and B*5801 specific epitopes. We analyzed the three B*57 and B*5801 immunodominant epitopes, TW10, (TSTLQEQIAW; HXB2 positions 241–249), ISW9 (ISPRTLNAW; HXB2 positions 147–155) and KF11 (KAFSPEVIPMF; HXB2 positions 162–172) for evidence of selection. Comparing sequences from the 21 individuals to the subtype C consensus sequence we calculated the proportions of non-synonymous (i.e. amino acid-changing) nucleotide differences that fell within or close to these epitopes (one flanking amino acid on either side of the epitope was included to allow for possible epitope processing escape mutations) using the SNAP program (www.hiv.lanl.gov). This analysis indicated that non-synonymous differences from the consensus subtype C sequences were more often associated with B*57 and B*5801 immunodominant epitopes for viruses with the A146X and/or T242N mutations than was the case for viruses without these mutations (p = 0.0010; Figure 1). These results suggests that these sequences had experienced greater selective pressure from the immune response around these immunodominant epitopes and supports our hypothesis that the women from which they were isolated were infected with CTL escape mutants that had arisen in HLA B*57/B*5801 positive individuals.

**Figure 2:**
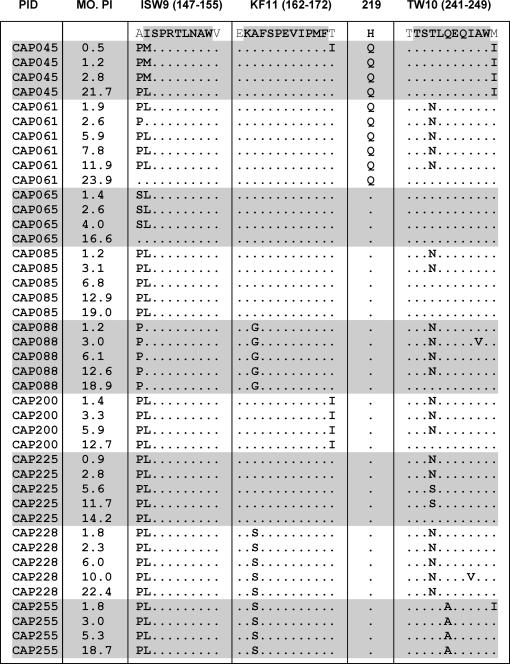
Sequence alignment of ISW9, KF11 and TW10 epitopes. Sequence changes of the 9 participants carrying the T242N and A146X mutations in B57/B5801-specific immunodominant epitopes over time showing reversion to consensus sequence at position 242. One flanking amino acid residue was included on either side of the epitope. The position of the H219Q compensatory mutation is indicated relative to the epitopes. MO. PI refers to months post-infection.

This analysis also revealed additional evidence of selection by B*57/B*5801 restriction. Apart from the potential immune evasion mutations at Gag positions 242 and 146, three sequences (CAP088, CAP228 and CAP255) also carried the well-studied A163X mutation in the HLA B*57/B*5801 restricted KF11 epitope (Figure 2) [12],[31],[32]. Viruses carrying the T242N and A146X escape mutants from two of the nine individuals (CAP045 and CAP061), also carried the H219Q compensatory mutation that has been shown to partially restore replicative fitness losses incurred by the T242N mutation [11],[21],[33].

### The T242N and A146X mutations revert over time

The T242N escape mutation is rare in chronically infected HLA-B*57/B*5801-negative individuals and has been known to revert rapidly in these individuals upon transmission from HLA-B*57/B*5801-positive donors [21]. Following limiting cDNA dilution and amplification, *Gag* sequences from the nine study participants infected with T242N/A146X mutants were analyzed over time to detect reversion mutations (Figure 2). Reversion of the T242N mutation was observed between six and 24 months post infection in five of the six (83%) individuals initially infected with viruses carrying this mutation (Figure 2). Reversion of the A146X mutation was only observed in two of the nine (22%) individuals infected with viruses carrying this mutation. These reversions were observed at 16 and 24 months post-infection. Sequences from the two individuals infected with viruses carrying the A163X mutation in the KF11 epitope did not show any reversion of this mutation.

**Figure 3:**
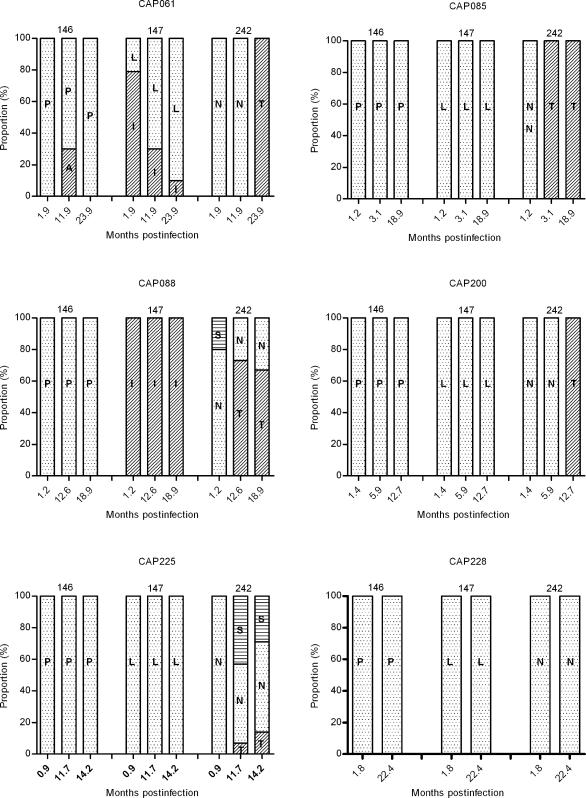
Proportion of HLA-B*57/5801-associated escape mutations at different time-points. Gag bulk PCR products were cloned and sequenced at three available timepoints as indicated on the x-axis. A median of 12 sequences (range 7–20) were analyzed per participant per time-point.

To investigate the proportion of T242N and wild-type variants in the six participants infected with T242N mutants, bulk PCR was performed and amplicons from three time points were cloned and sequenced. Although there was complete replacement of the escape mutation (N242) with the consensus amino acid (T242) in three participants (CAP061, CAP085 and CAP200), in another participant (CAP228) no reversion was observed (Figure 3). In the remaining two participants (CAP088 and CAP225), a mixed viral population consisting of both escape mutants and wild-type variants were detected at the final time point assayed, indicating that complete replacement of the escape mutant with the wild-type variant had not occurred. In CAP225 at 14.2 months, the escape mutation was detected in 8/14 clones (57%), the reversion intermediate, S242 was identified in 4/14 clones (29%), and T242 occurred in 2/14 clones (14%). In CAP088 the T242 wild-type was the dominant population member at both 12.6 and 18.9 months post infection with only, 3/11 and 4/12 of the sampled sequences at these respective timepoints displaying the N242 polymorphism. Reversion of the A146X mutation was only observed in one (CAP061) of the six individuals infected with viruses carrying the T242N mutation. However, although the wild-type A146 polymorphism was observed in 3/10 sampled sequences at 11.9 months post infection, it was not detectable amongst ten sequences sampled at 23.9 months post infection (Figure 3). The transience of this reversion indicates that in this participant at least, it may not have provided any substantial fitness advantage.

**Figure 4:**
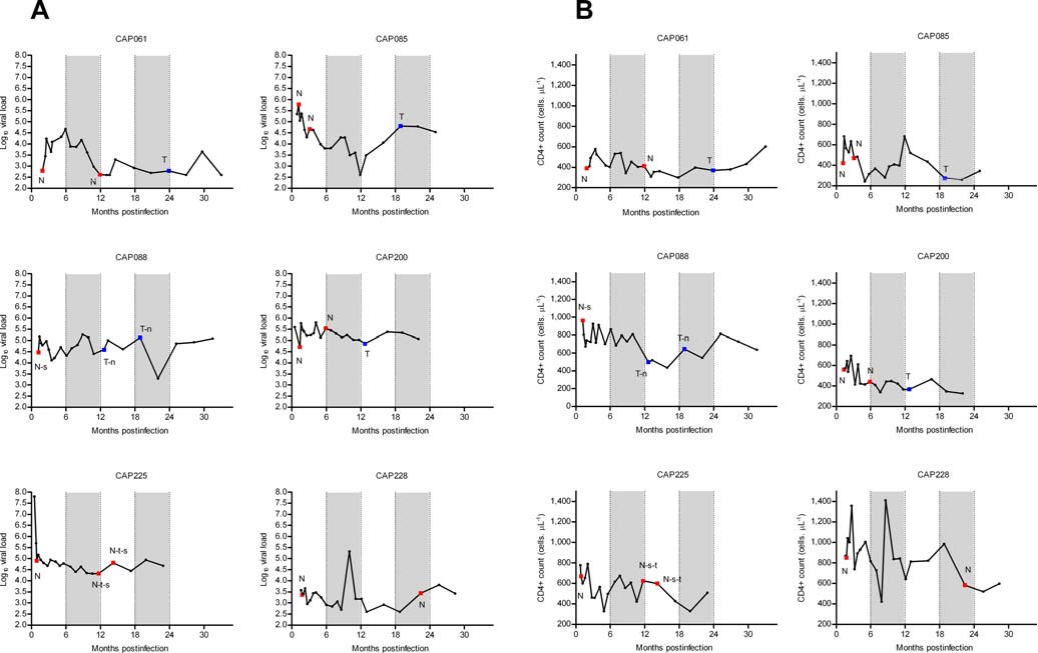
A) Viral load and B) CD4+ counts over time for the 6 participants infected with virus carrying the T242N mutation at enrolment. The amino acid residue at position 242 is indicated for the time-points cloned and sequenced. Red squares indicate N242 and blue squares indicate T242. Where there was more than one variant, the dominant residue is shown in upper case whereas lower case letters indicate the subdominant residues.

Viral load and CD4+ count dynamics were plotted over time to investigate whether reversion of T242N was associated with either increased viral loads or decreased CD4+ counts (n = 6 , Figure 4a and b). Overall, there was no significant change in geometric mean log viral loads or CD4+ counts between either 6–12 and 12–18 months post-infection, or 12–18 and 18–24 months post infection (p>0.2; Wilcoxon matched pairs test) (Table 2). However, one of the six study participants (CAP085) had an increase in log viral load of 1.05 and a corresponding decrease in CD4+ count of 209 cells.µl^−1^ between 12–18 and 18–24 months. The T242 reversion polymorphism was observed in this individual at 6.8 months post infection suggesting that the loss of viral control was not concomitant with reversion.

**Table 2:** Changes in viral load and CD4+ counts for the 6 participants infected with virus carrying the T242N mutation at enrolment.

PID	Δ log_10_ VL		Δ CD4+ count	
	6–12 and 12–18 months	12–18 and 18–24 months	6–12 and 12–18 months	12–18 and 18–24 months
CAP061	−0.64	−0.1	−111	53
CAP085	0.12	1.05	70	−209
CAP088	−0.02	−0.62	−290	110
CAP200	−0.08	0.09	11	77
CAP225	0.18	0.19	−71	−96
CAP228	−0.35	0.1	−40	4

Geometric means were calculated for viral loads and CD4+ counts between 6−12 months, 12−18 months and 18−24 months post infection and the differences in the geometric means over the three periods were tabulated.

These results confirm earlier reports [21] that the T242N mutations revert earlier than other HLA-B*57/B*5801 associated escape mutations and that A146X and other escape mutations persist as “footprints” of prior viral exposure to HLA-B*57/B*5801 alleles. In addition, reversion of T242N mutations to the wild-type consensus sequence does not have an obvious immediate impact on viral load.

### Phylogenetic clustering of A146X/T242N mutants

The 38% frequency of study participants infected with A146X/T242N mutants is higher than the 16.5% combined population frequency of HLA-B*57/B*5801 alleles [33]. However, this higher frequency is not inconsistent with all of the individuals infected with A146X/T242N mutants having received these viruses from HLA-B*57/B*5801 positive individuals. Given a sample of 21 individuals from this population we would expect between two and eleven individuals, to be either heterozygous or homozygous for at least one of these two alleles. We nevertheless sought to test whether the observed A146X and T242N mutations had all arisen independently. We analysed our 21 sequences together with 102 other sequences sampled from the same population and within five years of those sampled in our study [16]. We constructed a maximum likelihood tree from these sequences after discarding both nine potentially recombinant sequences and codons corresponding to known HLA-B*57/B*5801 associated escape mutations (Figure S1). Sequences carrying HLA-B*57/B*5801 associated escape mutations clustered significantly within this tree (p = 0.0157) indicating that there is a degree of epidemiological linkage amongst viruses carrying these mutations. This result also reiterates the notion that HLA-B*57/B*5801 associated mutations may persist for extended periods within circulating viruses.

### There is no enrichment of HLA types amongst study participants

Several other HLA alleles besides B*57 and B*5801 have also been associated with improved viral control [34]. It was possible that the reduced viremia and increased CD4+ counts, apparently associated with viruses carrying the A146X/T242N mutations, may have in fact been due to individuals infected with these viruses carrying HLA-alleles that are effective in controlling HIV. Compared to the remainder of the study population, we found no detectable enrichment of any alleles in the nine individuals infected with viruses carrying A146X/T242N mutations (data not shown), indicating that lower viremia and increased CD4+ counts were not obviously associated with over-representation of any one HLA allele.

### There is no difference in magnitude or breadth of responses to Gag between individuals infected with A146X/T242N^+^ and 146X/T242N^−^ variants

The number of Gag peptides recognised by CD8+ T-cells in ELIspot assays have been shown to be associated with viral control in subtype C HIV-1 infection [16]. To determine if the lower viremia observed in the 9 individuals infected with viruses carrying the putative transmitted CTL escape mutants was associated with the strength or breadth of Gag responses, IFN-gamma ELIspot responses were assessed at 9–15 weeks post-infection (Table 3). There was no significant difference between the group of individuals infected with the T242N/A146X mutants and that infected with the wild-type virus with respect to (1) the number of responders, (2) the magnitude of responses to Gag (as measured by total sfu/10^6^ PBMC for each Gag pool) and (3) the breadth of responses (as measured by the number of Gag pools recognized). Four out of nine individuals infected with T242N/A146X mutants had detectable responses to Gag with three recognizing single peptides (CAP085, 225, 228) (Table 3) and the fourth individual recognizing two peptides (CAP255). Of the 11 individuals infected with the wild-type variant, five responded to Gag, with two individuals recognising single peptides (CAP008, 084) and three targeting two peptides (CAP210, 256, 257) (Table 3).

**Table 3:** Interferon-gamma ELIspot responses for the 9/21 study participants who responded to Gag.

PID	HLA	Reactive peptide	SFU x 10^6^ (9–15weeks)
**CAP085**	**A3002 A3002 B0801 B4501 Cw0701 Cw1601**	**TGTEELRSLYNTVATLY**	a **138**
**CAP225**	**A0101 A3001 B4202 B8101 Cw0701 Cw1801**	**GATPQDLNTMLNTVGGH**	a **1058**
**CAP228**	**A2301 A2601 B4403 B5101 Cw0303 Cw0701**	**AFSPEVIPMFTALSEGA**	a **265**
**CAP255**	**A0301 A8001 B0801 B1807 Cw0202 Cw0702**	**IAPGQMREPRGSDIA** *	b **295**
	**A0301 A8001 B0801 B1807 Cw0202 Cw0702**	**WMTSNPPVPVGDIYWKRWI**	b **295**
CAP008	A2301 A2301 B0801 B1510 Cw0701 Cw1601	GKKHYMLKHLVWASREL	a1288
CAP084	A2902 A7401 B1503 B4407 Cw0210 Cw0701	TGTEELRSLYNTVATLY	b183
CAP210	A6802 A6802 B1510 B1510 Cw0304 Cw0304	NTMLNTVGGHQAAMQMLKDTINEEAAEWDRLHPV	b70
	A6802 A6802 B1510 B1510 Cw0304 Cw0304	GPKEPFRDYVDRFFKTLRAEQATQDVKNWMTDTL	a153
CAP256	A2902 A6601 B1503 B5802 Cw0401 Cw0602	PRTLNAWVKVIEEKAF	b58
	A2902 A6601 B1503 B5802 Cw0401 Cw0602	GATPQDLNTMLNTVGGHQAAMQMLK	b58
CAP257	A2301 A2902 B4202 B4403 Cw1701 Cw1701	GKKHYMLKHLVWASREL	a775
	A2301 A2902 B4202 B4403 Cw1701 Cw1701	MREPRGSDIAGTTSTL*	b115

In bold are the 4 individuals with the transmitted viruses carrying T242N/A146X mutations. Underlined are the known epitopes and their corresponding restricting HLA alleles.

*: No predicted epitope within the peptide matches the participant's HLA

a Derived from deconvoluting the pool matrix ELIspot response and subsequently confirming with single peptides

b Derived from deconvoluting the pool matrix ELIspot response only

No responses were detected to Gag fragments carrying the TW10 and ISW9 epitopes. Only one individual (CAP228) showed a response to a peptide overlapping with the KF11 epitope. The optimal epitope within this peptide restricted by the host HLA (HLA-A*2601) is EVIPMFSAL and the observed KF11 A163S escape mutation falls outside this epitope.

### Individuals infected with T242N/A146X mutants have lower viremia

Together these data support our hypothesis that the T242N and A146X mutations detected in viruses sampled from HLA-B*57/5801 negative individuals are genetic ‘footprints’ of prior passage through HLA-B*57/B*5801 positive donors. Initial identification of these sites was based on a naive scan of all Gag amino acid polymorphisms to identify those associated with high CD4+ counts and low viremia. The proposed mechanism whereby HLA-B*57/5801 individuals achieve good control of HIV replication is unclear, although targeting of the Gag TW10, ISW9 and KF11 epitopes is thought to contribute to this control [16],[21],[34]. Improved control of viral replication in such individuals is due, at least in part, to the fitness costs incurred by the T242N escape mutation in the TW10 epitope. We were therefore interested in determining the specific association of these escape mutations with viremia and CD4+ counts following their transmission to HLA-B*57/B*5801 negative recipients.

**Figure 5:**
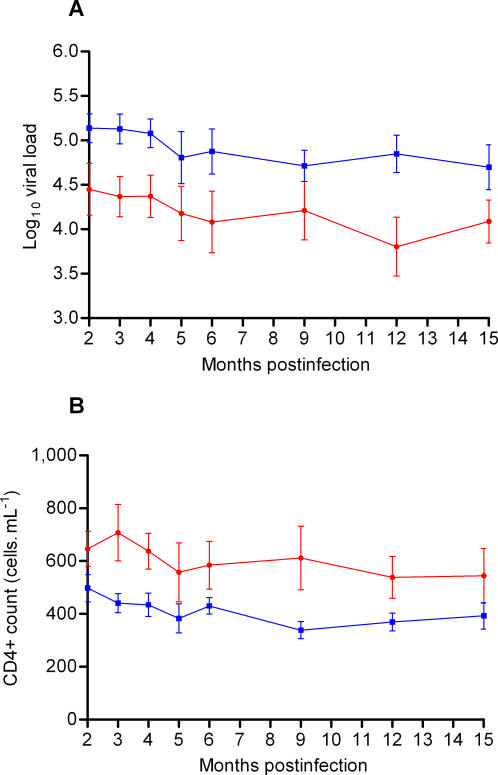
Mean and standard error of A) log viral loads and B) CD4+ counts over a 15 month period for the 21 study participants. The T242N/A146X+ participants are shown in red and the T242N/a146X- participants are shown in blue.

**Figure 6:**
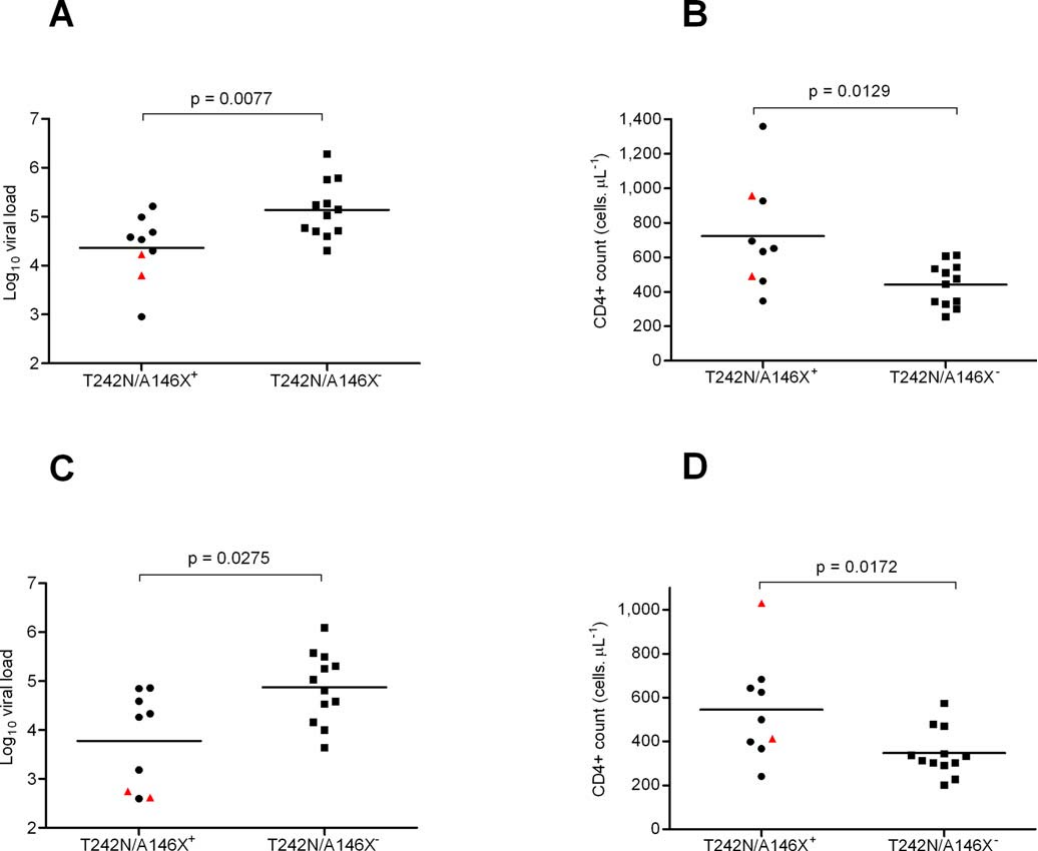
Viral Load and CD4+ counts of study participants grouped according to the presence or absence of the T242N and/or A146X mutations at enrolment. The 21 B57/B5801 negative individuals were grouped into those infected with viral strains comprising both the TW10 escape mutation and the ISW9 processing escape mutation (n = 9) and those that did not (n = 12). The viral load and CD4+ counts at 3 and 12 months post-infection were compared between these two groups. HLA-B*5801 positive individuals were excluded from the analysis. ▴ denotes viral loads and CD4+ counts for the two individuals infected with viruses carrying the H219Q compensatory mutation.

Clinical data was available from all individuals at 62 days post infection and we compared viral load and CD4+ dynamics up to 15 months post infection in the nine individuals infected with T242N/A146X mutants to those of the rest of the cohort (Figure 5a and b; Figure S2a and b). At all time points the mean log viral load and CD4+ count was lower in the individuals infected with the T242N/A146X mutants. We found that, relative to the rest of the study participants, individuals infected with the T242N/A146X mutants had significantly lower viral loads and higher CD4+ counts at three months post-infection (median log VL 4.53 vs. 5.09, p = 0.0077 and median CD4+ count 652.0 vs. 460.0, p = 0.0129), (Figure 6a and b). At 12 months post infection, these individuals also had significantly lower viral loads and higher CD4+ counts (median log VL 4.26 vs. 4.92, p = 0.0275 and median CD4+ count 499.0 vs. 322.5, p = 0.0172), (Figure 6c and d). This suggests that, in HLA-B*57/B*5801 negative individuals, HIV-1 variants carrying the Gag T242N and A146X mutations tend to be less pathogenic than those which do not carry the mutations. The H219Q mutation is a compensatory mutation reported to partially restore viral fitness [11]. However, we observed that the two individuals infected with H219Q mutant viruses tended to have lower viral loads within the T242N/A146X^+^ group (Figure 6a and c).

## Discussion

Examination of Gag sequences from acutely infected HLA-B*57/5801 negative women has revealed two polymorphisms, A146X and T242N, that are associated with lower viral loads and higher CD4+ counts in these woman up to a year post infection. As both polymorphisms have been previously identified as HLA-B*57/5801 immune evasion mutations we propose that they are probably genetic footprints of prior virus passage through HLA-B*57/5801 positive individuals. While HLA imprinting of circulating HIV sequences is an established phenomenon [35]–[38], our demonstration that such imprinting might enable better control of virus replication following transmission to HLA mismatched recipients is entirely novel.

While we do not provide definitive evidence that any of the studied viruses has been directly transmitted from HLA-B*57/B*5801 positive individuals, we have detected a strong signal of selection in the Gag HLA-B*57/B*5801 restricted epitope sequences of viruses carrying the A146X and T242N polymorphisms. This is suggestive of at least some of the viruses having been passaged through HLA-B*57/B*5801 positive individuals at some time in the past. Our speculation that the A146X/T242N mutants have been transmitted from either HLA-B*57 or HLA-B*5801 positive individuals is also consistent with previous reports dealing with the persistence of HLA-B*57/B*5801 associated immune evasion mutations. Two Gag mutations, H219X (X = Q, P or R) and the A146X polymorphism dealt with here, have been previously identified as relatively stable HLA-B*57/B*5801 genetic imprints on Gag [21]. These mutations appear to be epistatically associated with the T242N mutation but unlike the T242N mutation which reverts following transmission to HLA-B*57/5801-negative hosts, the H219X and A146X mutations are often maintained even in the absence of the selective forces exerted by these alleles [12],[21]. Whereas the H219X mutation partially alleviates the fitness deficit incurred by the T242N mutation [11], the A146X mutation has not been associated with any significant fitness loss [22]. Importantly, we detected the H219X mutation in two participants, one associated with a T242N mutation and the other was associated with the A146X mutation. In the latter case the presence of the H219X mutation suggests that this virus may have descended from a T242N mutant.

The possibility that most, if not all, of the six women infected with viruses carrying the T242N mutation were directly infected by HLA-B*57/5801-positive individuals is additionally supported by our observation that the mutation reverted in five of the individuals during the study period. It is, however, more uncertain whether viruses carrying the A146X mutation but not the T242N mutation were transmitted directly from HLA-B*57/5801-positive individuals. It cannot be discounted that the A146X mutation in these viruses might be a persistent imprint of a more distant passage through an HLA-B*57/5801 positive individual. While we have detected reversions of this mutation, it has persisted in the viruses infecting seven of the nine individuals studied here for more than two years and is clearly more stable than the T242N mutation. If the mutation had a reversion half-life of four or more years (as is suggested by our data) it would not be surprising if some of the A146X mutants we have studied had been serially transmitted two or more times since they first arose. Our detection of significant phylogenetic clustering of *gag* sequences carrying HLA-B*57/5801 associated escape mutations supports the notion that many of these mutations may persist for epidemiologically significant time periods in HLA-B*57/5801 negative individuals.

Reversion of the T242N escape mutation did not result in a concomitant increase in viral load. There is a relationship between viral load during primary infection and viral load set-point [39] and it is possible that these escape mutations provided a long term benefit by reducing viremia during acute infection. It is also possible that, amongst the viruses studied, reversion mutations in *gag* were not sufficient on their own to fully restore fitness and that there may have been other HLA-B*57/5801 associated escape and compensatory mutations elsewhere in their genomes that impacted on their fitness. Although there was only a marginal correlation between viral load and infection with variants harbouring both T242N and A146X mutations (p = 0.0733), this relationship was much stronger when individuals infected with only the A146X mutation were included in the analysis (p = 0.0275). This supports the existence of a network of B*57/5801 associated mutations that could contribute to viral control. Brockman *et. al*. [18] recently reported several novel compensatory mutations, associated with the T242N escape mutation, which correlated with higher viral loads. It might have been expected that the two individuals (CAP045 and CAP061) whose viruses had compensatory mutations would have higher viral loads as compared to those infected with the T242N/A146X-carrying viruses [11],[18]. Viral loads in these individuals were, in fact, lower. While our data suggests that, within the first year of infection, B*57/5801-negative individuals infected with viruses carrying these escape mutations have lower viral loads, the long-term impact on disease progression is unknown.

The T-cell responses determined in this study could not explain the differences in viral loads observed for the individuals infected with the escape mutants compared to those infected with the wild-type variants. The number of individuals that responded to Gag did not differ between the two groups and there were no significant differences in the magnitude or breadth of Gag IFN-gamma ELIspot responses between the two groups. However, it is likely that we are underestimating the T-cell responses due to the use of consensus based subtype C reagents compared to autologous peptides. In addition, experimental limitations could also be a contributing factor as the IFN-gamma ELIspot assay does not detect all T-cell responses.

We have therefore demonstrated that, during the acute phase of infection at least, individuals who are infected with viruses carrying markers indicative of previous selection in HLA-B*57/5801 positive individuals experience both significantly lower viral loads and higher CD4+ counts than individuals infected with viruses without these markers. These lower initial viral loads and higher CD4+ counts at the onset of infection may slow disease progression in these individuals. The possibility that an interacting network of attenuating mutations may be responsible for the lower viral loads experienced by people infected with viruses passaged through HLA-B*57/5801 positive individuals should be investigated further as the existence of such networks could profoundly influence our understanding of HIV pathogenesis. Current opinion is that first generation CTL based vaccines are likely to be only partially effective. Our study suggests that such vaccines should contain epitopes where escape is associated with a fitness cost to the virus as this might drive the attenuation of viruses in individuals who become infected despite vaccination.

## Methods

### Study Participants

Participants in this study are part of the CAPRISA 002 cohort investigating the role of viral and immunological factors in acute and early HIV-1 infections. The cohort includes high risk HIV negative women monitored monthly for recent HIV-1 infection using two HIV-1 rapid antibody tests and PCR (Roche Amplicor v1.5). HIV-1 infection was confirmed using an enzyme immunoassay (EIA) test. Women were enrolled in the present study within 3 months of infection from both the HIV negative cohort, and other seroincidence cohorts in Durban, South Africa. The timing of infection was estimated to be either at the midpoint between the last HIV-1 negative test and the first antibody positive test, or as 14 days where individuals were PCR positive-antibody negative. Samples were collected at enrolment, weekly for three weeks, fortnightly until 3 months, monthly until a year and quarterly thereafter. CD4+ T cells counts were assessed using a FACSCalibur flow cytometer and viral loads were measured using a COBAS AMPLICOR™ HIV-1 Monitor Test v1.5 (Roche Diagnostics). Plasma collected in EDTA was stored at −70°C until use. Written informed consent was obtained from all participants. This study received ethical approval from the University of KwaZulu-Natal, University of the Witwatersrand and University of Cape Town. All study participants in the cohort who had reached 12 months postinfection were included in this study, excluding 3 HLA-B*5801-positive individuals.

### RT-PCR and viral sequencing

RNA isolated from plasma samples using the Magna-Pure Compact Nucleic Extractor (Roche) was reverse transcribed using the Invitrogen Thermoscript Reverse transcription kit (Invitrogen) and the primer, *Gag* D reverse (5′-AAT TCC TCC TAT CAT TTT TGG-3′; HXB pos 2382-2402). Limiting dilution nested PCR was carried out by serial end-point dilution of the cDNA [40]. The first round PCR primers were Gag D forward (5′-TCT CTA GCA GTG GCG CCC G-3′; HXB pos 626–644) and Gag D reverse (5′-AAT TCC TCC TAT CAT TTT TGG-3′; HXB pos 2382–2402). The second round PCR primers were Gag A forward (5′-CTC TCG ACG CAG GAC TCG GCT T-3′; HXB pos 683–704) and Gag C reverse (5′-TCT TCT AAT ACT GTA TCA TCT GC-3′; HXB pos 2334–2356). PCR products were either directly sequenced or cloned using the -T Easy vector system (Promega). Sequencing was carried out using an ABI PRISM dye terminator cycle-sequencing kit (Applied Biosysytems) and the primers Gag A forward, Gag A reverse (5′-ACA TGG GTA TCA CTT CTG GGC T-3′; HXB pos 1282–1303), Gag B forward (5′-CCA TAT CAC CTA GAA CTT TGA AT-3′; HXB pos 1226–1246), Gag B reverse (5′-CTC CCT GAC ATG CTG TCA TCA T-3′; HXB pos 1825–1846), Gag C forward (5′-CCT TGT TGG TCC AAA ATG CGA-3′; HXB pos 1748–1768) and Gag C reverse for direct sequencing. For cloned sequences, only the Gag B forward and Gag B reverse primers were used generating p24 gag sequences. Sequences were assembled using the CAPRISA Assembly Pipeline tool (http://tools.caprisa.org/) and aligned using ClustalW (with default settings [41]).

### HLA typing

High resolution (four digit) HLA typing was performed on all participants. DNA was extracted from either PBMCs or granulocytes using the Pel-Freez DNA Isolation kit (Pel-Freez). HLA-A, -B and -C typing was performed by sequencing of exons 2, 3 and 4 using Atria AlleleSeqr kits (Abbott) and Assign-SBT 3.5 (Conexio Genomics). Any ambiguities resulting from either polymorphisms outside the sequenced exons or identical heterozygote combinations, were resolved using sequence-specific primers.

### IFN-γ Elispot assay

PBMC were prepared and HIV-1 specific T cell responses were quantified by gamma interferon (IFN-γ) Elispot assay [42]. Synthetic overlapping peptides(15- to 18-mer peptides overlapping by 10 amino acids) spanning the entire HIV-1 clade C Gag protein corresponding to the HIV-1 consensus C were used in the assay. T cell responses were derived using either a deconvoluted pool matrix approach or confirmed using individual peptides. The epitopes within peptides showing responses were predicted from the published epitopes on the Los Alamos HIV database (www.hiv.lanl.gov/content/immunology).

### Phylogenetic and recombination analyses

Phylogenetic trees were constructed using the maximum likelihood method implemented in PHYML [43] (100 full maximum likelihood bootstrap replicates, HKY model+gamma with four substitution rates and transition:transversion ratio determined from the data). Seven different recombination analysis methods implemented in RDP3 [44] were used, with default settings, to test for the presence of recombination amongst the 21 acute infection sequences and an additional 102 publicly available *gag* sequences sampled from a matched cohort. Evidence of phylogenetic clustering of viruses carrying particular Gag polymphisms was assessed using a permutation test (with 10000 iterations) implemented in RDP3 that is similar to that described in Poss *et al*. [45].

### Statistical analyses

Wilcoxon rank-sum tests were used to identify amino acid sites (encoded by the earliest *gag* sequences determined post infection) that were associated with low viral loads and high CD4+ counts at 12 months postinfection. These tests compared the median viral loads and CD4+ counts between groups of viruses with the consensus or an alternative amino acid at each site independently (without correction for multiple testing). Fisher's exact test was used to test each HLA allele for enrichment among individuals with either the A146X or T242N mutations and to test for associations between Gag ELIspot responses in the two groups (with and without T242N/A146X mutations). Changes in viral loads were tested using the Wilcoxon matched pairs test. Statistical tests were implemented in the R statistical computing environment [46] and GraphPad Prism 4.0 (GraphPad Software, Inc.).

### Nucleotide sequence accession numbers

Sequence data are available from GenBank under accession numbers EU347404–EU347714.

## Supporting Information

Figure S1 Maximum likelihood tree of HIV-1 subtype C gag sequences sampled in Durban South Africa. Whereas blue symbols represent sequences carrying nucleotide sequence polymorphisms characteristic of immune evasion mutations that occur in HLA-B*57/B*5801 positive individuals, red symbols indicate sequences without these polymorphisms. Blue bars to the right of the figure indicate clades in which sequences carrying the polymorphisms predominate. Sequences denoted with triangles are those determined in this study. Whereas branches labeled with filled circles have >50% bootstrap support, those labeled with open circles have between 25 and 50% bootstrap support.
(0.13 MB TIF)

Figure S2 A) Viral load kinetics and B) CD4+ count kinetics over a 15 month period. Red lines represent T242N/A146X+ individuals and blue lines represent T242N/A146X− individuals. Thick red and blue lines represent the medians for the T242N/A146X+ and T242N/A146X−, respectively.
(1.72 MB TIF)

Table S1 HLA alleles and viral loads for the 21 individuals in the study. Shown in bold are the 9 participants with the T242N/A146X escape mutations at enrolment.
(0.09 MB DOC)
