# Supplementary figures and images for: Transmission of HIV-1 CTL Escape Variants Provides HLA-Mismatched Recipients with a Survival Advantage
Source: PLoS Pathog. 2008 Mar 21;4(3):e1000033. doi: 10.1371/journal.ppat.1000033 (PMC2265427; doi:10.1371/journal.ppat.1000033)

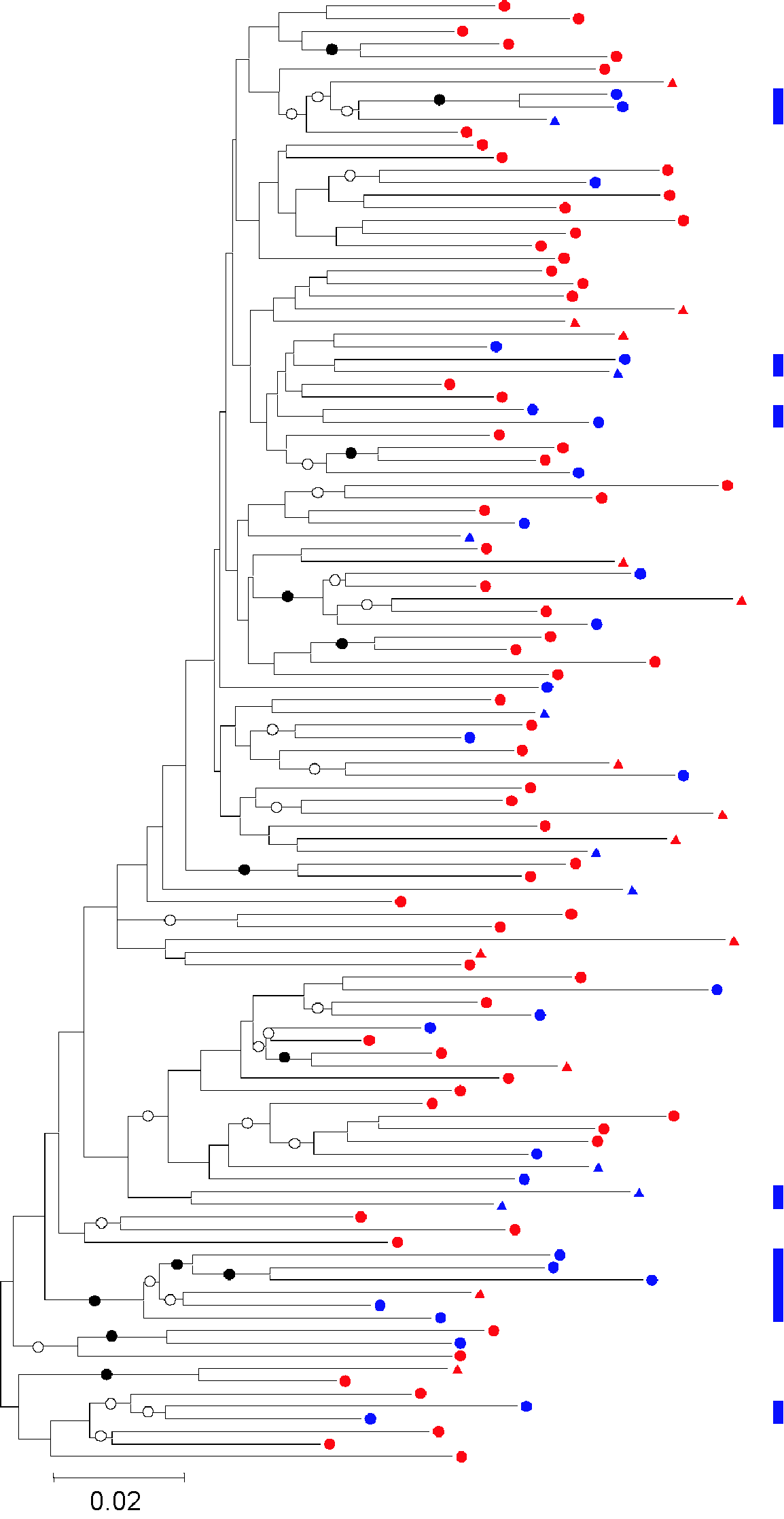

Supplement: Figure S1 — Maximum likelihood tree of HIV-1 subtype C gag sequences sampled in Durban South Africa. Whereas blue symbols represent sequences carrying nucleotide sequence polymorphisms characteristic of immune evasion mutations that occur in HLA-B*57/B*5801 positive individuals, red symbols indicate sequences without these polymorphisms. Blue bars to the right of the figure indicate clades in which sequences carrying the polymorphisms predominate. Sequences denoted with triangles are those determined in this study. Whereas branches labeled with filled circles have >50% bootstrap support, those labeled with open circles have between 25 and 50% bootstrap support. (0.13 MB TIF) [file ppat.1000033.s001.tif]

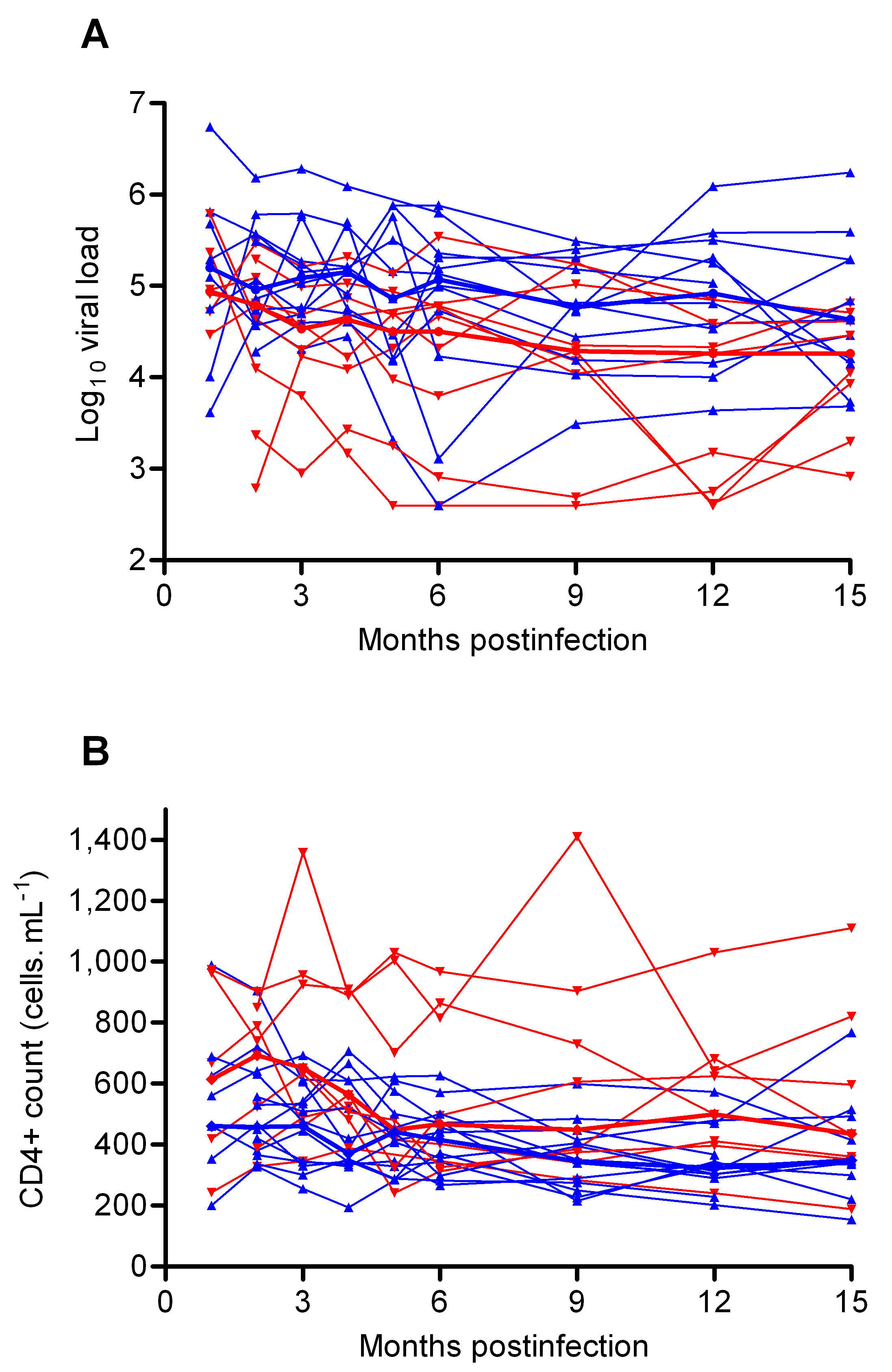

Supplement: Figure S2 — A) Viral load kinetics and B) CD4+ count kinetics over a 15 month period. Red lines represent T242N/A146X+ individuals and blue lines represent T242N/A146X− individuals. Thick red and blue lines represent the medians for the T242N/A146X+ and T242N/A146X−, respectively. (1.72 MB TIF) [file ppat.1000033.s002.tif]
