# Supplementary material for: Transmission of HIV-1 CTL Escape Variants Provides HLA-Mismatched Recipients with a Survival Advantage
Source: PLoS Pathog. 2008 Mar 21;4(3):e1000033. doi: 10.1371/journal.ppat.1000033 (PMC2265427; doi:10.1371/journal.ppat.1000033)
Supplement: Table S1 — HLA alleles and viral loads for the 21 individuals in the study. Shown in bold are the 9 participants with the T242N/A146X escape mutations at enrolment. (0.09 MB DOC) [file ppat.1000033.s003.doc]

**Supplementary Table 1: HLA alleles and viral loads for the 21 individuals in the study.** Shown in bold are the 9 participants with the T242N/A146X escape mutations at enrolment

| **PID** | **HLA** | | | | | |  | **Log_10_ viral load** | | | | |
| --- | --- | --- | --- | --- | --- | --- | --- | --- | --- | --- | --- | --- |
|  | **A** |  | **B** |  | **C** |  |  | **Enrolment** | **3*** | **6*** | **12*** | **15*** |
|  |  |  |  |  |  |  |  |  |  |  |  |  |
| **CAP045** | **3001** | **3002** | **1510** | **4201** | **0304** | **1701** |  | **5.37** | **3.80** | **2.60** | **2.75** | **3.93** |
| **CAP061** | **6602** | **6802** | **1401** | **4201** | **0802** | **1701** |  | **2.79** | **4.23** | **4.67** | **2.62** | **3.30** |
| **CAP065** | **2301** | **6802** | **1510** | **5802** | **0511** | **0611** |  | **4.96** | **4.53** | **-** | **4.85** | **4.71** |
| **CAP085** | **3002** | **3002** | **801** | **4501** | **0701** | **1601** |  | **5.79** | **4.30** | **3.80** | **2.60** | **4.05** |
| **CAP088** | **2901** | **6601** | **4501** | **5802** | **0602** | **0602** |  | **4.47** | **4.58** | **4.32** | **4.59** | **4.61** |
| **CAP200** | **0205** | **4301** | **1510** | **4101** | **0401** | **1701** |  | **4.72** | **5.21** | **5.54** | **4.86** | **-** |
| **CAP225** | **0101** | **3001** | **4202** | **8101** | **0701** | **1801** |  | **5.70** | **4.68** | **4.78** | **4.33** | **4.81** |
| **CAP228** | **2301** | **2601** | **4403** | **5101** | **0303** | **0701** |  | **3.58** | **2.95** | **2.91** | **3.18** | **2.92** |
| **CAP255** | **0301** | **8001** | **0801** | **1807** | **0202** | **0702** |  | **5.29** | **4.99** | **4.77** | **4.26** | **4.46** |
| CAP008 | 2301 | 2301 | 0801 | 1510 | 0701 | 1601 |  | 5.57 | 5.27 | 4.99 | 4.59 | 4.63 |
| CAP030 | 0201 | 3402 | 4403 | 4501 | 0401 | 1601 |  | 4.01 | 5.79 | 5.13 | 5.31 | 3.73 |
| CAP069 | 0301 | 2301 | 1503 | 5802 | 0511 | 0611 |  | 6.74 | 6.28 | 5.80 | 6.09 | - |
| CAP084 | 2902 | 7401 | 1503 | 4407 | 0210 | 0701 |  | 3.96 | 4.31 | 2.60 | 3.64 | 3.68 |
| CAP174 | 0301 | 7401 | 4901 | 5802 | 0602 | 0701 |  | 5.68 | 5.76 | 5.00 | 4.53 | 5.29 |
| CAP206 | 3204 | 7412 | 0702 | 4403 | 0202 | 0702 |  | 5.57 | 5.15 | 5.19 | 5.50 | 5.29 |
| CAP210 | 6802 | 6802 | 1510 | 1510 | 0304 | 0304 |  | 5.10 | 4.70 | 5.31 | 5.58 | 5.59 |
| CAP244 | 2301 | 3004 | 4403 | 5802 | 0401 | 0602 |  | 4.28 | 4.60 | 4.73 | 4.16 | 4.46 |
| CAP248 | 0205 | 2902 | 1401 | 1503 | 0202 | 0804 |  | 4.74 | 4.77 | 3.11 | 4.81 | 4.21 |
| CAP256 | 2902 | 6601 | 1503 | 5802 | 0401 | 0602 |  | 4.75 | 4.71 | 5.88 | 5.25 | 4.15 |
| CAP257 | 2301 | 2902 | 4202 | 4403 | 1701 | 1701 |  | 5.44 | 5.24 | 4.23 | 4.00 | 4.84 |
| CAP258 | 2301 | 2901 | 4101 | 4201 | 1701 | 1701 |  | 5.87 | 5.03 | 5.36 | 5.03 | - |
|  |  |  |  |  |  |  |  |  |  |  |  |  |
| **Median** |  |  |  |  |  |  |  | **5.10** | **4.71** | **4.78** | **4.59** | **4.46** |
|  |  |  |  |  |  |  |  |  |  |  |  |  |
| *Log_10_ viral loads at 3, 6, 12 and 15 months postinfection | | | | | |  |  |  |  |  |  |  |
